# Diversity Analysis of Sweet Potato Genetic Resources Using Morphological and Qualitative Traits and Molecular Markers

**DOI:** 10.3390/genes10110840

**Published:** 2019-10-24

**Authors:** Fabio Palumbo, Aline Carolina Galvao, Carlo Nicoletto, Paolo Sambo, Gianni Barcaccia

**Affiliations:** Department of Agronomy, Food, Natural resources, Animals and Environment (DAFNAE) University of Padova, Agripolis Campus, Viale dell’Università, 16-35020 Legnaro, Italy; fabio.palumbo@unipd.it (F.P.); aline.galvao@unipd.it (A.C.G.); paolo.sambo@unipd.it (P.S.); gianni.barcaccia@unipd.it (G.B.)

**Keywords:** *Ipomoea batatas*, genetic diversity, SSR markers, qualitative traits

## Abstract

The European Union (EU) market for sweet potatoes has increased by 100% over the last five years, and sweet potato cultivation in southern European countries is a new opportunity for the EU to exploit and introduce new genotypes. In view of this demand, the origins of the principal Italian sweet potato clones, compared with a core collection of genotypes from Central and Southern America, were investigated for the first time. This was accomplished by combining a genetic analysis, exploiting 14 hypervariable microsatellite markers, with morphological and chemical measurements based on 16 parameters. From the molecular analyses, Italian accessions were determined to be genetically very similar to the South American germplasm, but they were sub-clustered into two groups. This finding was subsequently confirmed by the morphological and chemical measurements. Moreover, the analysis of the genetic structure of the population suggested that one of the two groups of Italian genotypes may have descended from one of the South American accessions, as predicted on the basis of the shared morphological characteristics and molecular fingerprints. Overall, the combination of two different characterization methods, genetic markers and agronomic traits, was effective in differentiating or clustering the sweet potato genotypes, in agreement with their geographical origin or phenotypic descriptors. This information could be exploited by both breeders and farmers to detect and protect commercial varieties, and hence for traceability purposes.

## 1. Introduction

Sweet potato (*Ipomoea batatas* Lam.) is a root crop of the Convolvulaceae family, originating in Central and South America, which spread through the world with great ease due to its prominent productive efficiency. This crop plays a vital role in food production because it is one of the most important root and tuber crops in the world. Its ability to produce energy is very efficient and it can provide a significant quantity of protein and sugars per hectare in a short time [1].

In the European context, this crop had an enormous rise in consumption and, according to the Center for the Promotion of Imports from Developing Countries (CBI) [2], its importation has doubled in recent years. The European Union (EU) market for sweet potatoes has increased by 100% over the last five years (CBI, 2015), and sweet potato cultivation in southern European countries presents a new opportunity for the EU to exploit and introduce new genotypes. In Italy, it is considered a niche and ethnic product, and the recent immigration flow has created a market with increasing domestic demand [3] and many future opportunities for growth and profitability.

Despite the historical and commercial importance of sweet potatoes, to date, no study has investigated the origin, the conservation, or the genetic background of this species in Italy. On a larger scale, several works have been published [4,5,6,7,8] on the genetic characterization of sweet potato accessions, mainly to investigate the dispersal of New World sweet potato landraces from the center of origin (Tropical America, [9]). One of the main obstacles to the understanding of the dispersal dynamics of sweet potato throughout the world is probably the genetics of this hexaploid species (2*n* = 6x = 90) [10], which severely complicates any genomic approach. In particular, sweet potato is an allohexaploid species (AABBBB), most likely derived from the interspecific hybridization between a diploid and tetraploid species followed by chromosomal doubling [11,12]. As a consequence, its inheritance model is admixed, including both disomic (AA) and tetrasomic (BBBB) pairings. On the other hand, it must be recognized that this polyploidy could represent an important source of genetic diversity [13]. According to Silva Ritschel and Huamán [14], the vast genetic diversity that characterizes the sweet potato germplasm is also due to sexual reproduction (i.e., genetic segregation and recombination) and asexual propagation (i.e., fixation of specific genetic combinations), as well as to the exchange and introduction of plants from all over the world. This diversity provides a valuable source for potentially useful traits and allows plant breeders and farmers to adapt the crop to heterogeneous and changing environments [15]. The evolving climate conditions and the staggering expansion of the world population together represent pressing challenges for agriculture.

As already seen in other crops, morphological, agronomic, and molecular marker approaches are often used in combination to complement the information provided singularly in order to investigate the heterogeneity described in a species [16]. Molecular markers such as microsatellites or SSRs play a central role in the assessment and conservation of genetic diversity due to their efficiency, reliability, and reproducibility. Several studies based on the application of SSRs have recently attempted to monitor and prevent genetic erosion of local crop varieties in Italian scenarios [15,17,18,19]. Estimating the allelic dosage at each locus represents a critical question in polyploid species, even when using co-dominant markers such as microsatellites [20]. For this reason, as has already been done in previous studies [21,22,23], SSRs were scored as dominant markers and organized in binary matrixes, similarly to the process with AFLP or RAPD markers. According to Silva Ritschel and Huamán [14], morphological and chemical characterization is an indirect measure of population genetic diversity. Morphological markers for sweet potatoes are accessible and easy to use, making the technique one of the most used for this kind of analysis [24,25,26].

In this study, the geographical and genetic origins of the principal Italian sweet potato accessions were compared with a core collection of accessions from Central and South America for the first time. As has already been achieved in previous works on sweet potato [27,28], this was achieved by combining the high polymorphism and reproducibility of SSR markers with the high information value of strategic morphological and qualitative traits.

## 2. Materials and Methods

### 2.1. Plant Material

The cultivation was carried out at the experimental farm “L. Toniolo” of Padova University (45°21′ N; 11°58′ E; 8 m a.s.l.) in the 2016 spring/fall growing cycle. The propagation material used in the experiment was obtained from the germplasm bank of the Padova University (Table 1). The pedigree information of the plant materials derived from Central and Sothern America is unknown; as to the origin of Italian genotypes, we only know that they were introduced into Tuscany in 1630, cultivated until the end of the 1800s exclusively in botanical gardens, and only spread to the Northern Italy cultivation areas from 1880. In January 2016, sweet potato cuttings were produced in a glass greenhouse set with a temperature of 25 °C and 18 °C during the day and night, respectively. Thirty storage roots for each genotype, 40 mm to 80 mm in diameter, were placed in PVC pots (three roots per pot) filled with a peaty substrate (Klasmann Potgrond H) integrated with 20% perlite. In May, the cuttings were suitable for transplanting (0.30–0.35 m tall). Before transplanting, the soil was plowed and fertilized with 80, 70, and 210 kg ha^−1^ of N, P_2_O_5_, and K_2_O, respectively [29]. Cuttings were planted 0.10 m deep on the built-up rows, spacing the plants 0.35 m apart in the row and 0.80 m between rows. After transplanting, approximately 100 mL of water was provided for each cutting. The crop was irrigated three times during the growing cycle, at a rate of 30 mm m^−2^ for each irrigation. Sweet potatoes were harvested at the end of September 2016.

### 2.2. Molecular Analysis

#### 2.2.1. Genomic DNA Isolation 

Leaves were collected from 1 month old transplants, snap-frozen in liquid nitrogen upon harvesting, and stored at −20 °C until further processing. Approximately 100 mg of leaf tissue was employed for the isolation of genomic DNA using the DNeasy plant kit (Qiagen, Valencia, CA, USA), according to the manufacturer’s instructions. Extracted DNA samples were run on 0.8% agarose/1× TAE gel containing 1× SYBR Safe DNA stain (Life Technologies, Carlsbad, CA, USA) to evaluate their integrity. Both the purity and quantity were assessed with a NanoDrop 2000c UV-Vis spectrophotometer (Thermo Scientific, Pitsburgh, PA, USA).

#### 2.2.2. SSR Genotyping

For the SSR analysis, microsatellite markers belonging to 14 distinct genomic loci from both coding regions (EST-SSR) and non-coding regions (nSSR) were obtained from different sources [4,5,10,30] (Table 2). These markers were chosen due to the high polymorphism they showed in the reference studies. In order to evaluate the efficiency and the polymorphism degree of this SSR set, a preliminary test was performed using three different clones randomly chosen from the sweet potato collection, and each was analyzed in two biological replicates (i.e., two distinct plants for each clone). Moreover, each biological replicate was, in turn, analyzed in two technical replicates, to evaluate the reproducibility of the SSR.

The PCRs were carried out via the three-primer strategy reported by Schuelke [31], with a major modification first described by Palumbo et al. [32]; Instead of using only M13, three additional universal sequences (designated as PAN1, PAN2, and PAN3) were used to tag the 5’ end of the forward primer of each couple (colored sequences in Table 2) and adopted in combination with M13, PAN1, PAN2, and PAN3 fluorophore-labeled oligonucleotides. Fluorophores adopted were 6-FAM, VIC, NED, and PET, respectively. Due to the genetic complexity of the species and thus the possibility of obtaining up to six alleles per SSR locus, PCRs were performed in single reactions. Each reaction contained approximately 40 ng of genomic DNA template, 1× Platinum^®^ Multiplex PCR Master Mix (Applied Biosystems, Carlsbad, CA, USA), GC enhancer 10% (Applied Biosystems), 0.05 µM tailed forward primer (Invitrogen Corporation, Carlsbad, CA, USA), 0.1 µM reverse primer (Invitrogen Corporation), 0.23 µM universal primer (Invitrogen Corporation), and sterile water to volume. Amplifications were performed in a 96 well plate using a 9600 thermal cycler (Applied Biosystems), adopting the following conditions: after initial denaturation for 2 min at 95 °C, a touch-down PCR was undertaken with six cycles consisting of 30 s denaturation at 95 °C, 1 min annealing at 60 °C decreasing by 1.0 °C with each cycle and 30 s elongation at 72 °C; then 35 cycles at 95 °C for 30 s, 55 °C for 60 s, and 72 °C for 30 s. A final extension at 60 °C for 30 min terminated the reaction and filled in any protruding ends of the newly synthesized strands. The amplicons were visualized and quantified by agarose gel electrophoresis (2% agarose/1× TAE gel containing 1× Sybr Safe DNA stain (Life Technologies)), and the gel pictures were acquired with an UVITEC UV Transilluminator (Cambridge, UK) equipped with a digital camera. Subsequently, 10 ng of each PCR product was pooled and organized according to the four multiplexes reported in Table 2 and subjected to capillary electrophoresis on an ABI PRISM 3130xl Genetic Analyzer (Thermo Fisher) using LIZ500 (Applied Biosystems) as molecular weight standard and G5 (Applied Biosystems) as filter. Peak Scanner software v. 2.0 (Applied Biosystems) was used to determine the size of each peak, and each SSR was handled as a dominant marker. From the total of 14 SSR primer pairs initially selected for sweet potato genome analysis and tested for polymorphisms, Ib318 [4], J263 [5], and GDAAS0156 [10] showed weak resolution or screening errors and were excluded from our study.

#### 2.2.3. Marker Data Analysis

Data were coded as (0,1) vectors, where 1 indicated the presence and 0 the absence of a peak/allele at a specific position in the electropherogram. The polymorphic information content (PIC) of each SSR locus over its *n* marker alleles was computed as [33],

PIC = 1 − ∑p*_i_*^2^ (1)

where p*_i_* is the frequency of the marker allele *i*.

Genetic similarity between the clones was estimated by applying Dice’s coefficient [34] in all possible pairwise comparisons, and a triangular similarity data matrix was generated. The first two principal components of the matrix were thus computed through a principal coordinate analysis (PCoA). All calculations were conducted using NTSYS-pc v. 2.21q software [35]. Taking advantage of the genetic similarity data, PAST software v. 3.14 [36] was used to construct a dendrogram through the unweighted pair group arithmetic average (UPGMA) method and by applying the Dice’s coefficient [34]. To measure the stability of the computed branches, a statistical bootstrap analysis was conducted with 1000 resampling replicates. GenAlEX software v. 6.5 [37] estimated the number of observed alleles and the presence of private allele throughput in all the samples, purposely grouped as “Italian clones (*N* = 5)” and “foreign clones (*N* = 17)” according to their putative origin. Marker alleles were scored as “private” when shared by at least 60% of the individuals of one group and simultaneously absent from the other group. 

A Bayesian clustering algorithm implemented in STRUCTURE v. 2.2 software [38] was used to model the genetic structure of the considered *I. batatas* accessions’ haploid genotypes. The “admixture model” and “correlated allele frequencies model” were selected, because no prior knowledge about their origin was available (first model) and to guarantee the identification of a previously undetected correlation without affecting the results if no correlation existed [39] (second model). The number of founding groups ranged from 2 to 10, and 10 replicate simulations were performed for each K value, setting a burn-in of 2 × 10^5^ and a final run of 10^6^ Markov chain Monte Carlo (MCMC) steps [40]. Finally, the most likely estimation of K was decided by evaluating the rate of change in the log probability of data between successive K values (ΔK method), according to Evanno et al. [41]. In particular, one 2D Excel vertical histogram for each accession, conveniently divided into K colored segments, was used to represent the estimated membership in each hypothesized ancestral genotype. Each color correlated to a putative ancestor.

### 2.3. Morphological and Chemical Analyses

The morphological characterization was performed in August 2016. Root shape, root skin color, root flesh color, and the general outline of the leaf were scored according to the morphological descriptors available from International Board for Plant Genetic Resources (IBPGR) [42]. All the morphological traits considered for each genotype were transformed into numbers using the CIP scale [42] in order to process them with statistics. Three biological replicates were performed for the chemical analyses in order to recover representative data about the sweet potato samples.

#### 2.3.1. Extraction of Phenols for Analysis

Freeze-dried samples (1 g) were extracted in methanol (20 mL) with an Ultra Turrax T25 (IKA-Labortechnik, Staufen, Germany) at 1018 rpm until a uniform consistency was achieved. Samples were filtered (589 filter paper; Whatman, Germany) and appropriate aliquots of extracts were assayed by the Folin–Ciocalteu (FC) method [43] for total phenolic (TP) content, and by the ferric reducing antioxidant power (FRAP method) for antioxidant activity [44]. For HPLC analyses, extracts were further filtered with cellulose acetate syringe filters (0.45 μm porosity).

#### 2.3.2. Determination of TP Content by the FC Assay

The TP content was determined according to the FC assay, using gallic acid as a calibration standard and a UV-1800 spectrophotometer (Shimadzu, Columbia, MD, USA). The FC assay was carried out by putting 200 μL of sweet potato extract into a 10 mL test tube, followed by the addition of FC reagent (1 mL). The mixture was vortexed for 20–30 s and 800 μL of filtered 20% sodium carbonate solution was added 1–8 min after the FC reagent addition. The mixture was then vortexed for 20–30 s (time 0). The absorbance of the colored reaction product was measured at 765 nm after two hours at room temperature. The TP content in the extracts was calculated from a standard calibration curve obtained with different concentrations of gallic acid, ranging from 0 to 600 μg mL^−1^ (coefficient of determination: *r*^2^ = 0.9992). The results have been expressed as mg gallic acid equivalent (GAE) kg^−1^ dry weight.

#### 2.3.3. Determination of Total Antioxidant Activity by FRAP

Freshly prepared FRAP reagent contained 1 mmol L^−1^ 2,4,6-tripyridyl-2-triazine and 2 mmol L^−1^ ferric chloride in 0.25 mol L^−1^ sodium acetate (pH 3.6). A methanol extract aliquot (100 μL) was added to the FRAP reagent (1900 μL) and accurately mixed. Absorbance was determined at 593 nm after leaving the mixture at 20 °C for 4 min. The calibration was performed with a standard curve (0–1200 μg mL^−1^ ferrous ion) (coefficient of determination: *r*^2^ = 0.9985) obtained by the addition of freshly prepared ammonium ferrous sulfate. FRAP values were calculated as μg mL^−1^ ferrous ion (ferric reducing power) from three determinations and have been reported as mg kg^−1^ of Fe^2+^ (ferrous ion equivalent) of dry matter.

#### 2.3.4. Quantitative Determination of Ions by IC and Organic Nitrogen

For the estimation of anions and cations, a freeze-dried sample (200 mg) was extracted in water (50 mL) and shaken at 150 rpm for 20 min. Samples were filtered in sequence through filter paper (589 Schleicher), and the extracts were further filtered through cellulose acetate syringe filters (0.20 mm) before analysis by ion chromatography (IC). The IC was performed using an ICS-900 ion chromatography system (Dionex Corporation) equipped with a dual piston pump, a model AS-DV autosampler, an isocratic column at room temperature, a DS5 conductivity detector, and an AMMS 300 suppressor (4 mm) for anions and CMMS 300 suppressor (4 mm) for cations. A Dionex Ion-Pac AS23 analytical column (4 × 250 mm) and a guard column (4 × 50 mm) were used for anion separations, whereas a Dionex IonPac CS12A analytical column (4 × 250 mm) and a guard column (4 × 50 mm) were used for cation separations. The eluent consisted of 4.5 mM sodium carbonate and 0.8 mM sodium bicarbonate at a flow rate of 1 mL min^−1^ for anions, and 20 mM metansolfonic acid for cations at the same flow rate. Chromeleon 6.5 chromatography management software was used for system control and data processing. Anions and cations were quantified following a calibration method. Dionex solutions containing seven anions and five cations at different concentrations were taken as standards, and the calibration curves for anions and cations were generated with concentrations ranging from 0.4 mg L^−1^ to 20 mg L^−1^ and from 0.5 mg L^−1^ to 50 mg L^−1^ of standards, respectively. The Kjeldahl method (ISO 1656) was used for organic nitrogen.

#### 2.3.5. Brix Content

Approximately 0.5 mL of defrosting liquid of the product was used for the determination of the Brix content, carried out using a Hanna Instruments HI 96801 portable digital refractometer.

#### 2.3.6. Starch

Starch analysis was performed by chromatographic analysis according to AOAC Official Method 996.11 (University of Florida, IFAS, Bulletin 339-2000 “Starch Gelatinization & Hydrolysis Method” Boehringer Mannheim, Starch determination, cat. N° 207748).

#### 2.3.7. Quantitative Determination of Sugars by HPLC

Freeze-dried sweet potato root samples (0.2 g) were homogenized in demineralized water (20 mL) with an Ultra Turrax T25 until a uniform consistency was achieved at 1018 *g*. Samples were filtered in sequence through filter paper (589; Schleicher), and the extracts were further filtered through cellulose acetate syringe filters (0.45 mm) and analyzed by HPLC. The liquid chromatography apparatus utilized in this analysis was a Jasco X.LC system consisting of a model PU-2080 pump, a model RI-2031 refractive index detector, a model AS-2055 autosampler, and a model CO-2060 column. ChromNAV Chromatography Data System software was used for analysis of the results. The separation of sugars was achieved on a Hyper-Rez XP Carbohydrate Pb^++^ analytical column (7.7 × 300 mm; Thermo Scientific, Waltham, MA, USA), operating at 80 °C. Isocratic elution was effected using water at a flow rate of 0.6 mL min^−1^. D-(+)-glucose, D-(−)-fructose, and maltose were quantified by a calibration method. All standards utilized in the experiments were accurately weighed and dissolved in water; the calibration curves were generated with concentrations ranging from 100 to 1000 mg L^−1^ of standards.

### 2.4. Statistical Analysis

Chemical and morphological data were finally used to construct a constrained UPGMA dendrogram using PAST software v. 3.14 [36], applying the Euclidean similarity index and keeping the position of the samples fixed throughout the tree according to the clustering resulting from the SSR-based dendrogram. To measure the stability of the branches, a statistical bootstrap analysis was conducted with 1000 resampling replicates.

The complete set of data for each variety was used for random combinations using the bootstrap method. For each variety, a set of 1000 combinations was produced, and the data were analyzed by the PCoA procedure using the software Statgraphics Centurion 18.1.06 (Statgraphics Technologies, Inc.). All qualitative trait data were processed by ANOVA and, in case of significant differences, average values were separated by Tukey HSD test (CoStat 6.400–CoHort Software, CA, USA).

## 3. Results and Discussion

Overall, 117 marker alleles were detected in 11 SSR loci analyzed throughout the accession pool, ranging from a minimum of 6 (J206A) to a maximum of 16 (GDAAS0757), with an average number equal to 10.5 per locus (Table 3 and supplementary Table S1). According to Botstein et al. [33], all examined marker loci were found to be highly informative and variable across the accessions, with a mean PIC value equal to 0.79, spanning from 0.61 (J206A) to 0.93 (GDAAS0615), as reported in Table 3. Since private polymorphisms are recognized as an efficient molecular tool for food traceability, the presence of marker alleles able to discriminate the accessions according to their putative origin was investigated. As many as 58 out of the total 117 marker alleles scored (Table 3, blue boxes) were exclusively identified only within the foreign accessions pool, but only two of them, i.e., loci IBSSR04 and J116a (Table 3, underlined percentages) were scored in at least 60% of the accessions. In contrast, six marker alleles were exclusively associated with the Italian pool (Table 3, red boxes) and only one, i.e., locus IBSSR27, was found in at least 60% of the Italian sweet potatoes (Table 3, underlined percentage).

**Figure 1:**
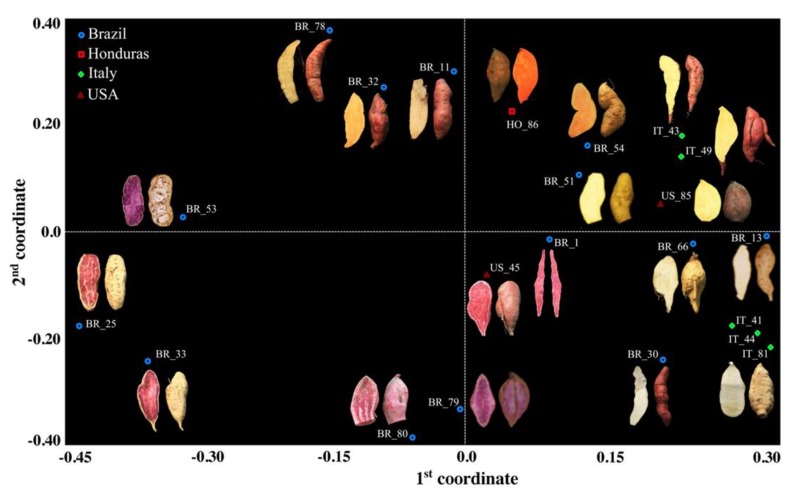
Principal coordinate analysis (PCoA) of the sweet potato core collection based on molecular markers: two-dimensional centroids derived from the genetic similarity estimates computed among accessions in all possible pairwise comparisons using the whole SSR marker data set. The first two coordinates were able to explain 54% of the total variation, accounting for 31% and 23% of the total, respectively. Four different colors have been used to distinguish the accessions based on their geographical origin: blue = Brazil, red = Honduras, green = Italy, and brown = USA.

According to the genetic similarity matrix calculated in all possible pairwise comparisons among the 22 accessions, Dice’s coefficient ranged from 0.28 (between BR_78 and BR_30) to 0.97 (between IT_41 and IT_44, Table S2). US_45 resulted to be the most divergent genotype: the average genetic similarity value calculated against the rest of the accessions was as low as 0.41, highlighting a clear-cut differentiation from the rest of the pool. The mean genetic similarity value calculated among the five Italian accessions was 0.70. The same value calculated in all pairwise comparisons within the Brazilian accessions was considerably lower (0.54), consistent with the great morphological variability observed within the South American core collection. The accession BR_66 was the most closely related to the Italian clones, scoring an average genetic similarity value of 0.70 and a maximum of 0.74 when compared with IT_44. From the principal coordinate analysis (PCoA), the first coordinate separated a group composed of eight Brazilian entries (namely, BR_25, BR_53, BR_33, BR_32, BR_11, BR_78, BR_79, and BR_80) from a group including all of the Italian clones (Figure 1).

**Figure 2:**
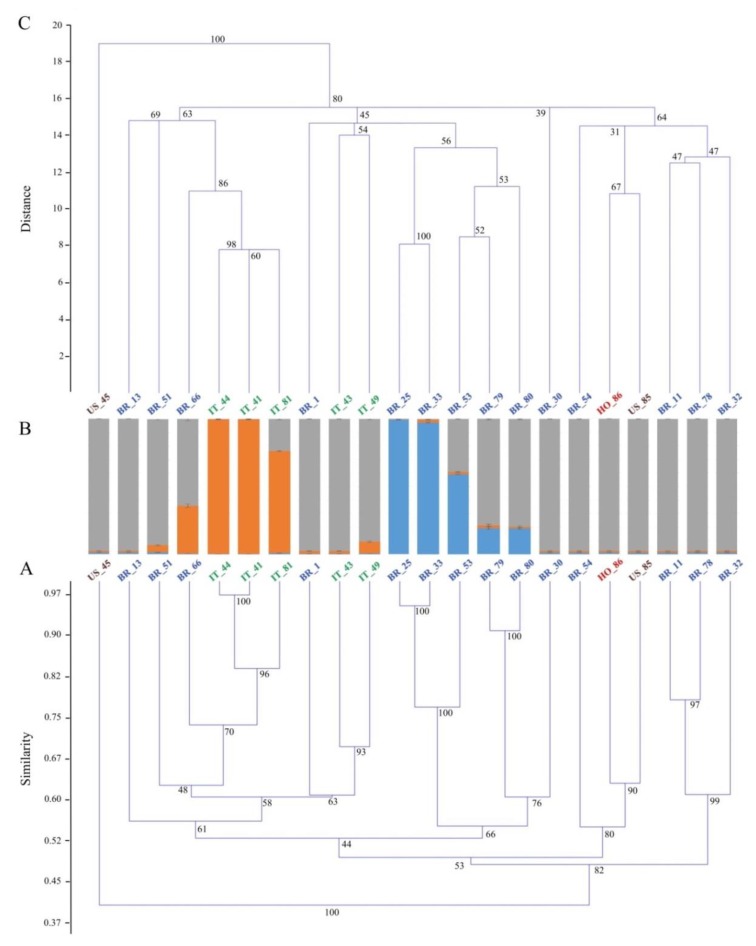
Genetic structure analysis of the sweet potato core collection: (**A**) The unweighted pair group method with arithmetic average means (UPGMA) tree of the genetic similarity estimates computed among pairwise comparisons of sweet potato accessions using the whole simple sequence repeat (SSR) marker data set, with nodes of the main subgroups supported by bootstrap values. The color scheme for this figure is the same as that used in Figure 1 (Blue = Brazil, red = Honduras, green = Italy, and brown = USA). (**B**) Population genetic structure of a core collection of *N* = 22 sweet potato accessions estimated using 11 microsatellite markers. Each sample is represented by a vertical bar partitioned into *K* = 3 colored segments representing the estimated membership. The proportion of ancestry (%) is reported on the ordinate axis, and the identification number of each accession is indicated below each histogram. (**C**) The unweighted pair group method with arithmetic average means (UPGMA)-constrained tree was built by applying the Euclidean similarity index and using the morpho-qualitative measurements of a subset of the *I. batatas* core collection. The positions of the samples throughout the dendrogram were kept fixed according to those ones resulting from the dendrogram in Figure 2A, and the bootstrap values supporting each node of the main subgroups were calculated.

The first principal coordinate also underlined the separation of the two US accessions (US_45 and US_85), according to their contrasting phenotype and in agreement with their low genetic similarity value (0.38). The main result of the variation explained by the second principal coordinate was the split of three Italian accessions showing a cream skin color (namely, IT_44, IT_81, and IT_41) from two Italian accessions distinguishable for their pink skin color (IT_49 and IT_43). This finding was supported not only by a contrasting phenotype, but also by a relatively low estimate of mean genetic similarity (0.60) calculated between these two groups, suggesting a different origin of the Italian accessions. The UPGMA analysis confirmed the sharp detachment, already seen in the genetic similarity matrix (Table S2), of US_45 from the rest of the genotypes (Figure 2A), supported by a bootstrap value of 100.

Four main clustering patterns already highlighted with the PCoA (Figure 1) were also observed throughout the dendrogram (Figure 2 panel A), with bootstrap values always higher than 90%. In detail, BR_79 and BR_80 grouped together and scored the 91% of similarity; BR_33, BR_25, and BR_53 shared a mean genetic similarity value of 82%; IT_44, IT_41, and IT_81 showed, on average, 88% the genetic similarity and, finally, BR_11 grouped with BR_78 according to a genetic similarity of 78%. In particular, it is worth highlighting that although the five Italian accessions were all part of the same macrobranch of the tree, they subclustered into two groups, according to what was observed in the PCoA analysis. In fact, in the PCoA, the second coordinate alone explained 23% of the total variation, clearly separating IT_44, IT_41, and IT_81 from a second group including IT_43 and IT_49 (Figure 1). A noteworthy consideration involving BR_66 is the close association between this Brazilian accession and the first group of Italian accessions in both the PCoA and the UPGMA tree, with a similarity value of 0.74. 

The ΔK criterion suggested by Evanno et al. [41] gave the highest value for the SSR analysis at three groups (for *K* = 3, ΔK resulted 58.53, Figure S1). According to this estimate, the whole group putatively originated from three ancestral genotypes (indicated in grey, orange, and blue in Figure 2B). One vertical histogram for each accession, conveniently divided into *K* = 3 colored segments, has been used to represent the estimated membership in each hypothesized ancestral genotype, and 90% was the threshold set for the admixed ancestry (Figure 2B). This finding seems to be in agreement with those reported by Roullier et al. ([45] Figure 2 in Appendix S1) and Wadl et al. [8], both supporting the *K* = 3 in the sweet potato clones from tropical America. Overall, one of the three ancestors (the one in grey) was predominant: 13 samples from USA, Honduras, Italy, and Brazil showed a membership to this ancestral genotype higher than 95%, suggesting a probable common origin. Five accessions (BR_66, IT_81, BR_53, BR_79, and BR_80) were admixed and one of the three ancestors (the one in grey) was always recurrent; interestingly, no example of hybridization between the other two ancestors (blue and orange, Figure 2) was observed.

In detail, two of three Italian accessions characterized by a cream skin color (IT_44 and IT_41) and strictly associated with the same branch of the UPGMA tree (similarity = 0.97) also shared the same marker allele cluster, both with accession scores of individual membership higher than 99%. Although these two Italian accessions were collected in two different areas, these results suggest a case of synonymy and we may suppose that the same genotype is cultivated in different regions with a different name. IT_81 and BR_66, although counted as admixed, shared the same ancestral genotype as IT_44 and IT_41, with percentages of 75% and 35%, respectively. Considering the PCoA and the UPGMA tree, it is probable that all four shared the same ancestral genotype (the one indicated in orange), and it is not to be ruled out that the three Italian accessions derived from BR_66. However, it is possible that BR_66 has undergone continuous events of hybridization with individuals descending from the “grey” progenitor, which would explain the admixed pattern of this Brazilian accession. In contrast, the three Italian accessions, whose memberships ranged from 75% (IT_81) to 99% (IT_44 and IT_41) may have preserved their "ancestral purity" thanks to repeated crosses with accessions of the same lineage or to asexual propagation breeding schemes. A second cluster included BR_25 and BR_33 (membership > 97%) and, to a lesser extent, BR_53 (membership = 59%), according to their sharp detachment from the rest of the pool highlighted by the PCoA analysis. The remaining accessions, including two Italian accessions (IT_49 and IT_43), were all part of a third cluster, except for BR_79 and BR_80, which were admixed (Figure 2B). The Euclidean-index-based UPGMA dendrogram emphasized the clear-cut detachment of US_45 from the rest of the pool (Figure 2C), as previously established by the SSR-based UPGMA (Figure 2A). In this case, the bootstrap support was 100%. The other samples were all clustered in three main branches, with a bootstrap value of 80%. In the first branch, BR_79, BR_80, BR_33, BR_25, and BR_53 clustered together with IT_43, IT_49, and BR_1. This finding was different from that observed in the SSR-based UPGMA tree (Figure 2A), where these two subgroups clustered separately, and it was not consistent with the PCoA analysis and the morphological data (Figure 1). Nevertheless, it must be noted that in the morphological and chemical-marker-based dendrogram (Figure 2C), the bootstrap support of this specific node was quite low (45%). Moreover, despite the different clusterization highlighted in the two UGPMA trees (Figure 2A,C), the two subgroups maintained the same composition. In particular, all of the samples in the first subgroups (BR_79, BR_80, BR_33, BR_25, and BR_53) were characterized by a typical purple color of flesh that was not detected in other samples. This observation, along with a full or partial membership to the same cluster (Figure 2 panel B), reinforces the hypothesis of a common ancestor for this group of samples, in agreement with their geography. Regarding the second subgroup (IT_43, IT_49, and BR_1), it is very hard to make hypotheses about its origin, although genetic data would presuppose a certain degree of kinship with the other three Italian accessions. Considering the second main branch of the Euclidean-distance-based UPGMA, two considerations are needed. First, IT_44, IT_41, IT_81, and BR_66 grouped together according to all the previous analyses (bootstrap support 86%). The fact that the genetic and the morpho-agronomic markers led to identical results may suggest that this first group of Italian genotypes was descended from BR_66, as initially predicted on the basis of their highly similar morphology (Figure 1). All the accessions were also analyzed by means of morpho-agronomic qualitative markers (Table 4).

**Figure 3:**
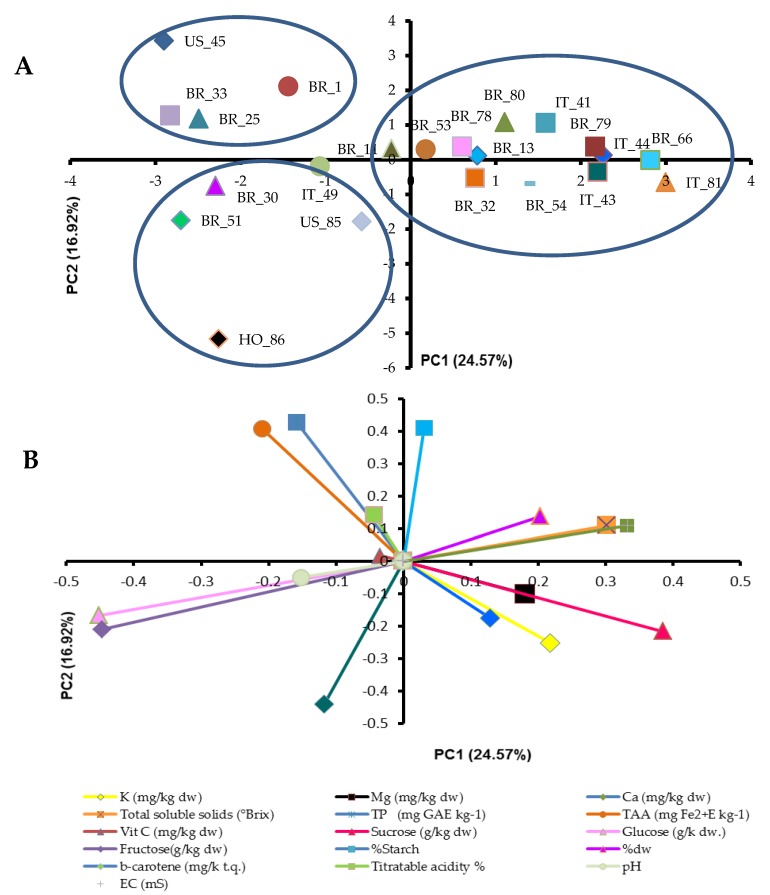
Principal components analysis (PCA) of the sweet potato core collection based on qualitative traits: (**A**) Score plot of the first two principal components (PC1 and PC2) for the 22 sweet potatoes. (**B**) Eigenvectors of the variables measured for the first two principal components. Loadings (eigenvalues) for the first and second principal components were equal to 25% and 17%, respectively. TP: total phenols; TAA: total antioxidant activity; dw: dry weight.

In addition to genetic characteristics, quality traits are generally preferred for nutritive value and market demand [46,47,48]. With regard to the qualitative aspects, the dataset obtained for the different parameters is summarized in Figure 3 by multivariate analysis.

The first identified the different genotypes positioned on the basis of the measured qualitative characteristics, whereas the second vectorially highlighted the main qualitative traits that determined the genotype positioning. The results obtained from this elaboration allowed us to identify interesting cues regarding grouping of the different genotypes according to their qualitative peculiarities. In particular, it was possible to group the 22 genotypes into three macrogroups (A, B, and C). Group A comprised those genotypes characterized by a high anthocyanin content in both the skin and the flesh (US_45, BR_1, BR_33, and BR_25). The genotypes belonging to this group were characterized by high total antioxidant capacity and high total polyphenol content (Figure 3). The anthocyanins belong to the antioxidant family, in particular to the polyphenols, and have positive effects on human health [49]. Anthocyanin composition was determined in purple-fleshed sweet potatoes [50,51], highlighting that cyanidin and peonidin glycosides acylated with phenolic acids were the primary anthocyanin components. The second group, B, comprised genotypes phenotypically characterized by high beta-carotene content, thus featuring orange pulp, and by high simple sugar concentrations (BR_51, BR_30, IT_49, US_85, and HO_86). Carotenoids are secondary plant compounds that form lipid-soluble yellow, orange, and red pigments. Carotene-rich vegetables are associated with decreased risk of chronic diseases related to vision, skin, infection, and reproduction, in addition to being active oxygen species scavengers [52]. The most abundant carotenoid in sweet potato roots is usually β-carotene, which comprises more than 77% of total carotenoid content and can reach more than 99% in sweet potatoes characterized by orange flesh [53]. The colored genotypes considered in this experiment were characterized by a β-carotene content ranging from 23.8 to 811 µg g^−1^. This range was wider than those measured by Simonne et al. [54] (1–190 µg g^−1^) and by Grace et al. [50] (1–253.3 µg g^−1^) in several sweet potato varieties. Finally, group C contained genotypes characterized by different flesh colors, but they shared a high content of sucrose and minerals (BR_32, BR_54, IT_43, and IT_81) or a high percentage of dry matter, soluble solids, and starch (BR_53, BR_78, BR_80, IT_41, BR_79, and BR_66). The high presence of starch and sucrose makes these varieties particularly sweet after slow cooking processes (i.e., boiling, oven, steam), following the production of maltose [3,55]. Genotypes belonging to group A and B may be more suitable for faster cooking methods (i.e., frying) because they are characterized by less starch, and they are more appealing for the consumer from a color point of view.

As shown from the comparison between grouping results of the core collection of sweet potato accessions (see Figure 1 and Figure 3), some inconsistencies arose between the clustering based on molecular markers and that derived from qualitative parameters. This was understandable since both the molecular markers and qualitative parameters evaluated in this study not only represented a subset of the evaluable genotypic and phenotypic traits, but also because they did not show any known linkage. Consequently, a full overlap with what was detected by the principal components based on qualitative traits is not possible, as demonstrated by the centroids derived from the principal coordinates of molecular markers. An example of this behavior can be found between IT_43 and IT_49: these genotypes were grouped together based on molecular markers, whereas they differed according to the qualitative profiles (e.g., the former scored a higher concentration of potassium, whereas the latter a higher content of simple sugars).

## 4. Conclusions

We investigated, for the first time, the genetic structure and qualitative composition of the principal Italian sweet potato clones, along with their relatedness to a core collection of accessions from Central and Southern America. It is worth mentioning that the sweet potato accessions analyzed in this study represent the most cultivated clones in Italy and, to the best of our knowledge, no other locally adapted varieties are commercially available to Italian farmers. In fact, these materials, grown mainly in the Veneto region, are known to possess a high adaptation to the natural and anthropological environment in which they have been introduced and are still cultivated.

From the molecular analyses, Italian accessions were sub-clustered into two groups and were found to be genetically very similar to the South American germplasm. This finding was also supported by the morphological and chemical measurements affecting their principal qualitative traits.

Summarizing our results and considering both the morphological and qualitative and the genetic–molecular data, it is evident how the combination of these two different approaches was very effective in differentiating or clustering the different clonal genotypes, as expected by their geographical origin or their phenotypic characteristics. Moreover, because the molecular and the chemical results were often comparable, it was possible to make robust speculations on the common origins of sweet potato accessions. The experiment demonstrated not only a good relationship between genetic and morphological and qualitative aspects, but also allowed us to indirectly highlight the good level of adaptation of South American genotypes to European conditions. This last information allows us to suggest that breeders use South American germplasm, characterized above all by colored pulp, for the constitution of new genotypes in Europe, useful for the renewal and innovation of the European market as well as for providing new opportunities for farmers. On the whole, this information could be exploited by both breeders and farmers to detect and protect commercial varieties, and hence to certify the genetic identity of their propagation materials and overall quality of their food derivatives.

## Figures and Tables

**Table 1:** List of sweet potato genotypes used.

Genetic Material	Plant Type	Country of Origin	Flesh Color	Skin Color	Root Shape
BR_1	Extremely spreading	Brazil	Purple	Dark purple	Elliptical
BR_11	Spreading	Brazil	Cream	Pink	Round elliptical
BR_13	Extremely spreading	Brazil	White	Cream	Elliptical
BR_25	Semi-erect	Brazil	Purple	Cream	Long oblong
BR_30	Spreading	Brazil	White	Pink	Long irregular
BR_32	Semi-erect	Brazil	Pale orange	Pink	Oblong
BR_33	Semi-erect	Brazil	Purple	Cream	Oblong
BR_51	Extremely spreading	Brazil	White	Cream	Long elliptical
BR_53	n.a.	Brazil	Purple	White	Oblong
BR_54	Extremely spreading	Brazil	Intermediate orange	Yellow	Elliptical
BR_66	n.a.	Brazil	White	White	Irregular
BR_78	Semi-erect	Brazil	Cream	Pink	Long irregular
BR_79	Spreading	Brazil	Purple	Pink	Obovate
BR_80	n.a.	Brazil	Purple	Dark purple	Obovate
IT_41	Semi-erect	Italy	Cream	Cream	Long irregular
IT_43	Spreading	Italy	Pale yellow	Pink	Obovate
IT_44	Semi-erect	Italy	White	Cream	Elliptical
IT_49	Semi-erect	Italy	Pale yellow	Pink	Round elliptical
alIT_81	Erect	Italy	Cream	Cream	Obovate
US_45	Semi-erect	USA	Purple	Dark purple	Long oblong
US_85	n.a.	USA	Pale yellow	Cream	Round elliptical
HO_86	n.a.	Honduras	Deep Orange	Purple red	Round elliptical

n.a.: not available.

**Table 2:** List of SSR marker loci used in this study along with their basic information. For each SSR region, locus name, melting temperature (Tm), primer sequences, microsatellite motif, number of alleles according to the reference study, amplicon length in base pairs (bp), microsatellite type (including *n*SSR = SSR from non-coding regions and EST−SSR = SSR from coding regions), multiplex organization (for the simultaneous run of PCR products from different SSR marker loci in capillary electrophoresis) and the sources from which the SSR data were obtained are reported. Colored bases at the 5’ end of each forward primer represent four universal sequences (designated as M13, in blue, PAN1, in green, PAN2, in yellow and PAN3, in red) complementary to as many fluorophore-labeled oligonucleotides (fluorophores adopted were 6-FAM, VIC, NED and PET).

Locus Name	Tm (°C) *	Primer Sequence (5’–3’)	SSR Motif	Alleles	Length (bp)	Type	Multiple ×	Source
IBSSR04	62	GAGGTAGTTATTGTGGAGGACCTCCTTTGCCTCCTTTCATGC	(GA)11	7	216	nSSR	1	[30]
	62	CCTTGCTCCCCATTTTCTTCTTG						
J263	62	GGAATTAACCGCTCACTAAAGCTCTGCTTCTCCTGCTGCTT	(AAC)6	7	156–171	nSSR	1	[5]
	61	GTGCGGCACTTGTCTTTGATA						
J544b	61	TTGTAAAACGACGGCCAGTAGCAGTTGAGGAAAGCAAGG	(TCT)6	8	174–194	nSSR	1	[5]
	59	CAGGATTTACAGCCCCAGAA						
Ib318	60	GAGGTAGTTATTGTGGAGGACAGAACGCATGGGCATTGA	n.a.	5	125-135	nSSR	1	[4]
	60	CCCACCGTGTAAGGAAATCA						
Ib-255F1	61	GGAATTAACCGCTCACTAAAGCGTCCATGCTAAAGGTGTCAA	n.a.	8	210-245	nSSR	2	[4]
	59	ATAGGGGATTGTGCGTAATTTG						
Ib297	59	GAGGTAGTTATTGTGGAGGACGCAATTTCACACACAAACACG	(CT)13	24	129–167	nSSR	2	[5]
	60	CCCTTCTTCCACCACTTTCA						
Ib286	62	TTGTAAAACGACGGCCAGTAGCCACTCCAACAGCACATA	n.a.	10	90–122	nSSR	2	[4]
	57	GGTTTCCCAATCAGCAATTC						
IbS11	58	GGAATTAACCGCTCACTAAAGCCCTGCGAAATCGAAATCT	(TTC)10	13	218–248	nSSR	3	[5]
	61	GGACTTCCTCTGCCTTGTTG						
J116a	57	GAGGTAGTTATTGTGGAGGACTCTTTTGCATCAAAGAAATCCA	(CCT)7	15	187–227	nSSR	3	[5]
	60	CCTCAGCTTCTGGGAAACAG						
J206A	59	TTGTAAAACGACGGCCAGTATCAGGGAGAGAGGACAGTAA	(GAT)6	9	103–121	nSSR	3	[5]
	57	TAGGCAAACCATAAACAGAGA						
GDAAS0615	56	TGTAGAAAGACGAAGGGAAGGCCACATACAGACTACAACTTAC	(GA)10	7	230	EST-SSR	4	[10]
	57	GGAGGAGCGTATTATGAACA						
GDAAS0757	56	TTGTAAAACGACGGCCAGTGAGATGATGACGATAGTGTTG	(GAA)11	9	293	EST-SSR	4	[10]
	56	GGAAGATTCATTGGCAGAAG						
IBSSR27	56	GGAATTAACCGCTCACTAAAGGTGTTTATCACATCGTTTTCTG	(TA)6(CA)16	9	149	nSSR	4	[30]
	55	GGCTCGTACAATTTTCAAAG						
GDAAS0156	54	GAGGTAGTTATTGTGGAGGACTCCAAATACCATACCCAAC	(TC)10	8	118	EST-SSR	4	[10]
	55	CGCTTTCAAATAGAATCGTC						

* Tm of the forward primers does not take into account the tail sequence.

**Table 3:** Marker allele size, overall allele frequencies (F.o.), allele frequencies in the foreign accessions (F.f.), allele frequencies in the Italian accessions (F.i.), and polymorphism information content (PIC) values are reported for the 11 SSR loci. The blue boxes highlight private marker alleles found only in the foreign accessions, while red boxes denote marker alleles detected only in the Italian accessions. Percentages underlined designate private marker alleles shared by at least 60% of the foreign samples, as well as private marker alleles shared by at least 60% of the Italian clones.

IBSSR04					J544b					Ib-255F1					Ib297					Ib286					ibS11				
Size	F.o.	F.f.	F.i.	PIC	Size	F.o.	F.f.	F.i.	PIC	Size	F.o.	F.f.	F.i.	PIC	Size	F.o.	F.f.	F.i.	PIC	Size	F.o.	F.f.	F.i.	PIC	Size	F.o.	F.f.	F.i.	PIC
216	9%	12%	0%	0.75	184	91%	88%	100%	0.63	233	18%	24%	0%	0.87	137	9%	6%	20%	0.87	97	5%	6%	0%	0.77	224	9%	12%	0%	0.87
218	18%	24%	0%		178	18%	24%	0%		237	23%	24%	20%		147	5%	6%	0%		99	5%	6%	0%		227	55%	41%	100%	
220	59%	59%	60%		189	18%	24%	0%		239	5%	6%	0%		149	59%	53%	80%		101	18%	24%	0%		230	18%	24%	0%	
222	45%	65%	0%		192	18%	24%	0%		243	5%	6%	0%		151	14%	18%	0%		105	32%	41%	0%		233	9%	6%	20%	
224	18%	12%	40%		195	59%	47%	100%		251	50%	53%	40%		155	64%	71%	40%		107	14%	18%	0%		236	55%	53%	60%	
226	68%	71%	60%		198	82%	76%	100%		253	45%	53%	20%		159	14%	18%	0%		109	100%	100%	100%		239	32%	29%	40%	
228	50%	47%	60%		209	82%	76%	100%		255	73%	65%	100%		161	55%	41%	100%		113	77%	71%	100%		242	27%	18%	60%	
230	82%	76%	100%							257	14%	18%	0%		163	64%	65%	60%		115	45%	41%	60%		245	64%	71%	40%	
										259	64%	53%	100%		167	27%	35%	0%		121	59%	71%	20%		248	41%	53%	0%	
										261	9%	12%	0%		169	5%	0%	20%		123	9%	12%	0%		251	18%	24%	0%	
										263	32%	41%	0%		171	9%	0%	40%							254	59%	59%	60%	
										265	27%	35%	0%		175	5%	6%	0%							257	9%	12%	0%	
										267	9%	0%	40%		177	27%	35%	0%							260	14%	18%	0%	
															187	32%	41%	0%							263	9%	12%	0%	
**J116a**					**J206A**					**GDAAS0615**					**GDAAS0757**					**IBSSR27**									
**Size**	**F.o.**	**F.f.**	**F.i.**	**PIC**	**Size**	**F.o.**	**F.f.**	**F.i.**	**PIC**	**Size**	**F.o.**	**F.f.**	**F.i.**	**PIC**	**Size**	**F.o.**	**F.f.**	**F.i.**	**PIC**	**Size**	**F.o.**	**F.f.**	**F.i.**	**PIC**					
194	23%	29%	0%	0.69	118	82%	76%	100%	0.61	214	36%	29%	60%	0.93	272	18%	24%	0%	0.89	139	24%	29%	0%	0.88					
200	77%	71%	100%		124	55%	59%	40%		220	14%	18%	0%		281	23%	12%	60%		155	5%	6%	0%						
203	14%	12%	20%		127	100%	100%	100%		226	9%	12%	0%		284	18%	24%	0%		161	14%	0%	60%						
206	91%	88%	100%		130	5%	6%	0%		230	68%	76%	60%		290	9%	12%	0%		163	10%	12%	0%						
209	32%	41%	0%		133	59%	53%	80%		232	5%	6%	0%		293	14%	6%	40%		165	95%	88%	100%						
212	55%	47%	80%		136	9%	12%	0%		234	9%	12%	0%		296	41%	53%	0%		167	10%	12%	0%						
215	82%	76%	100%							236	23%	24%	20%		299	5%	6%	0%		169	5%	6%	0%						
218	9%	12%	0%							246	9%	12%	0%		302	50%	41%	80%		171	5%	0%	20%						
221	50%	65%	0%							248	14%	18%	0%		305	27%	35%	0%		177	29%	29%	20%						
										250	18%	18%	20%		308	5%	6%	0%											
										256	5%	0%	20%		311	14%	12%	20%											
															314	36%	29%	60%											
															317	95%	100%	80%											
															320	9%	12%	0%											
															323	27%	24%	40%											
															326	18%	6%	60%											

**Table 4:** Qualitative measurements performed on the sweet potato accessions.

Genotype	K (mg/kg dw)	Mg (mg/kg dw)	Ca (mg/kg dw)	Total Soluble Solids (°Brix)	TP (mg GAE kg^−1^ fw)	TAA (mg Fe^2+^E kg^−1^ fw)	β-Carotene (mg/kg fw)	Vit C (mg/kg dw)	Sucrose (mg/kg dw)	Glucose (mg/k dw)	Fructose (mg/kg dw)	Starch (%)	Dry Matter (%)
US_45	7381 klm	2426 bcde	1810 abc	9 bc	6442 a	6706 a	n.d.	658 c	36430 l	35280 cd	33561 b	81 a	36 ab
BR_79	12151 b	1841 def	2481 ab	12 a	2875 bc	2563 cd	n.d.	2967 ab	109542 defg	12903 jklm	11357 fghi	71 ab	37 ab
BR_80	5907 n	2181 cde	2405 ab	11 ab	2369 bc	2599 cd	n.d.	3966 ab	93156 fghi	9487 klmn	8501 fghi	74 ab	43 a
BR_33	6828 lm	1460 ef	1582 c	8 c	1753 c	2095 cd	n.d.	3887 ab	37464 l	26310 de	29451 bcd	83 a	36 ab
BR_25	7074 lm	1580 ef	2104 ab	9 bc	3240 bc	3578 bc	n.d.	2988 ab	55692 kl	31784 cd	31623 bc	72 ab	36 ab
BR_53	8965 g	1732 def	2308 ab	11 ab	3358 bc	2869 cd	n.d.	1933 bc	116776 cde	21199 efg	22460 de	70 b	34 b
BR_13	7328 klm	2640 abc	1981 abc	9 bc	1839 c	2210 cd	n.d.	2950 b	106360 def	8561 lmn	6028 hij	65 bc	32 b
BR_1	5316 no	1484 ef	1908 abc	11 ab	4425 ab	4651 b	n.d.	4682 a	81680 hij	21667 efg	15816 ef	69 b	36 ab
BR_66	10647 de	2270 bcd	1795 bc	13 a	2078 bc	2156 cd	23.8	2305 b	149539 a	6364 mn	6803 ij	64 bc	42 a
IT_44	8045 ij	2658 abc	1922 abc	9 bc	875 c	834 e	n.d.	1690 bc	109205 def	4396 lmn	3763 j	79 ab	37 ab
IT_41	10626 de	2156 cde	1709 bc	10 b	1595 c	1335 de	n.d.	2112 b	99035 fg	8673 lmn	7189 hij	83 a	37 ab
IT_81	12176 b	2697 abc	1925 abc	10 b	1067 c	862 e	n.d.	2544 b	124873 bcd	5007 n	3671 j	72 ab	36 ab
BR_51	9403 g	2056 bcd	1816 abc	9 bc	1186 c	2019 cd	34.2	3856 ab	113505 def	46730 a	46568 a	70 b	30 b
IT_43	15129 a	1729 cde	1954 abc	11 ab	2085 bc	1906 de	n.d.	1867 bc	144240 ab	12253 ijklm	10429 fgh	75 ab	34 b
IT_49	7855 ijk	2302 bcd	1563 c	10 b	1453 c	1607 de	208	1389 bc	92224 ghi	36301 bc	24108 cd	80 a	30 b
BR_30	6766 lm	2118 cde	1848 bc	8 c	1657 c	1743 de	90.8	1427 bc	68226 jk	45548 a	33478 b	70 b	31 b
BR_54	6666 m	2619 abc	2624 a	9 bc	1317 c	1577 de	571	2285 b	138292 bc	13282 hijk	10709 fgh	78 ab	37 ab
HO_86	11914 bc	2192 bcd	2282 ab	9 bc	1189 c	1179 de	512	2477 b	95520 fgh	42100 ab	45446 a	52 c	36 ab
US_85	8970 g	2023 cde	2264 ab	8 c	720 c	799 e	n.d.	1595 c	69641 jk	25645 def	30837 bc	60 c	36 ab
BR_32	8096 hi	1645 def	2298 ab	11 ab	1551 c	1516 de	811	4709 a	138295 bc	17282 ghij	14933 efg	72 ab	32 b
BR_11	10431 f	1452 f	1763 bc	9 bc	2477 bc	1073 e	n.d.	3087 ab	88841 ghi	19294 efgh	14664 efg	72 ab	35 b
BR_78	6850 lm	2352 bcd	1808 bc	10 b	2119 bc	1809 de	n.d.	1256 c	100268 efg	17742 fghi	13919 fg	70 b	42 a

Within each parameter, values without common letters significantly differed at *P* < 0.05 according to Tukey’s HSD test (n.d., not determined).

## Supplementary Materials

The following are available online at https://www.mdpi.com/2073-4425/10/11/840/s1, Figure S1: Definition of the number of ancestral sweet potato genotypes. Mean ΔK is calculated as L”(K)/(SD(L(K))) following Evanno et al. (Evanno et al. 2005), where mean LnP(D) ± SD over 10 runs is a function of K, being L’(K) = ΔLnP(D), Table S1: SSR data recorded for the different sweet potato genotypes, Table S2: Genetic similarity matrix for the sweet potato accessions.
