# Supplementary figures and images for: Diversity Analysis of Sweet Potato Genetic Resources Using Morphological and Qualitative Traits and Molecular Markers
Source: Genes (Basel). 2019 Oct 24;10(11):840. doi: 10.3390/genes10110840 (PMC6895877; doi:10.3390/genes10110840)

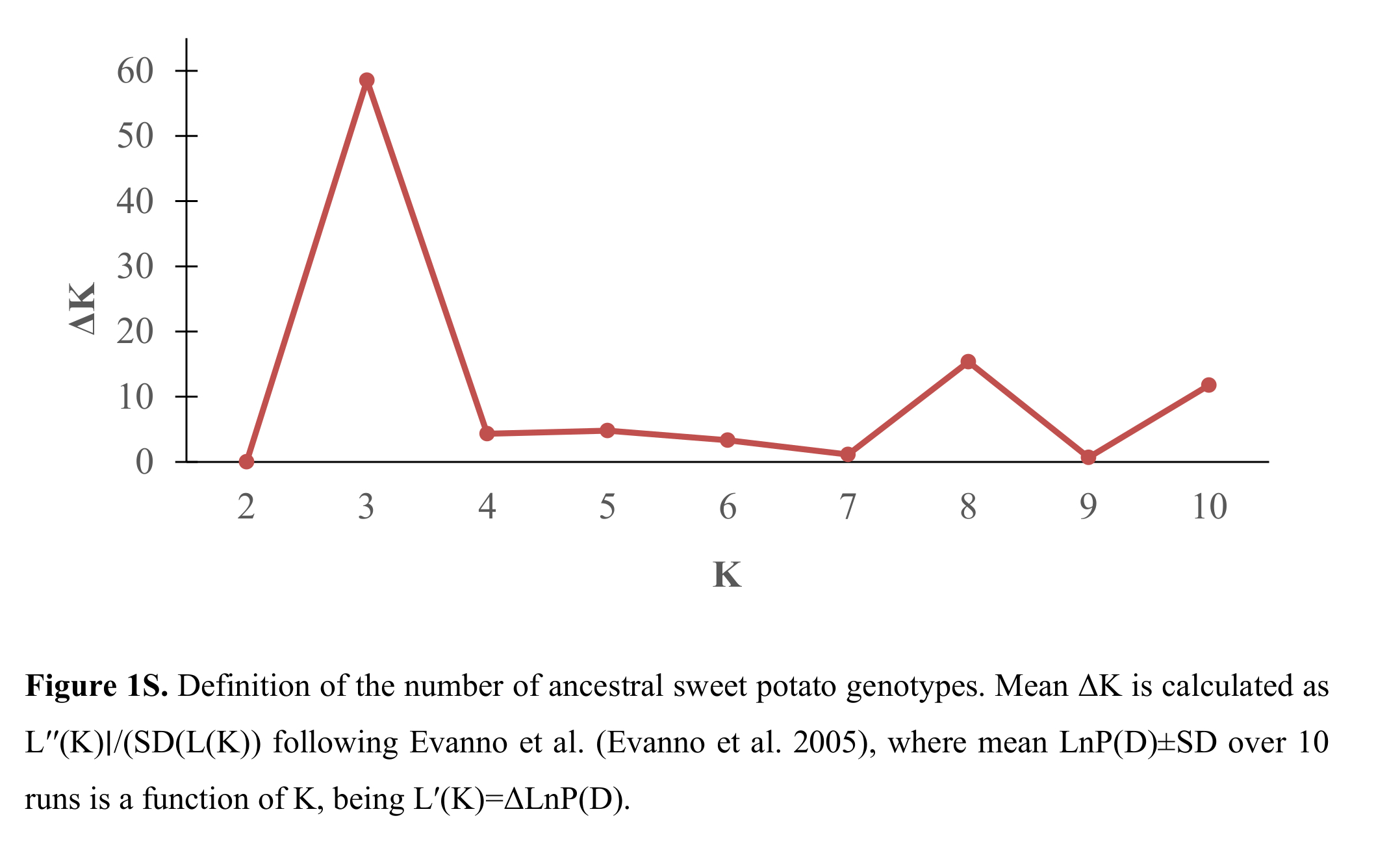

Supplement: Supplementary file 1 [file genes-10-00840-s001.zip › Figure S1.jpg]
